# On Acclimation

**Published:** 1850-08

**Authors:** Philip C. Williams

**Affiliations:** Winchester, Virginia


### MEDICAL EXAMINER,

AND

### RECORD OF MEDICAL SCIENCE.

NEW SERIES.—NO. LXVIII.—AUGUST, 1850.

### ORIGINAL COMMUNICATIONS.

On Acclimation. *An Inaugural Essay presented for the Degree of Doctor of Medicine in the University of Pennsylvania.* By Philip C. Williams, M. D., of Winchester, Virginia.

Acclimation—the result of the various modifications in the human system, when it is subjected to the influence of a climate different from that to which it has been accustomed—is a theme of deep interest.

It is one, the proper appreciation of which would prevent or greatly mitigate many of the diseases to which man is now subjected, in his migrations for the sake of travel, commerce, and the pursuit of science. The question assumes still greater importance when viewed in its relation to colonization, or the settlement of large bodies of men in regions where the climate is often so different from that of their own native land. Within the limits of the United States alone, the changes thus effected by the continual movement of the people, from the old to the new States and territories, invest the problem with a peculiar and home interest.

It seems to be an established law of nature, that the constitution of man must be modified by the atmospheric influences with which he is surrounded, and that it must carry the impress of the climate which he inhabits.

While we admit the difficulty of accounting for all the distinctive traits that characterize the chief races of mankind, we cannot deny that many of the modifications in their external appearance, and physiological actions, are attributable to the influence of climate. Did the narrow limits of an inaugural essay permit, I might, to substantiate this view, repeat the affirmative opinions of Hippocrates, Montesquieu, and Cabanis. The "Father of Medicine" was the first to point out the controlling influence of climate, as he did so clearly in his essay on "Waters, Airs, and Places," when he compared the people of Europe with those of Asia, and attributed the superiority of the former over the latter, to a purer and cooler atmosphere. Montesquieu appeals to history to show that, in the various struggles among rival candidates for imperial sway at Rome, he who secured the assistance of the European legions was sure of success. General history, in its narratives of the overthrow of empires, kingdoms, and nations, points to the same conclusion.

There are many circumstances which combine to produce these modifications of climate, and these changes in the human system; such as the thermometric and hygrometric states of the atmosphere; the elevation above the level of the sea; the prevalence of, and the exposure to, particular winds, &c.

Let us, for a moment, examine the first of these, viz., the effects of temperature. The range of temperature compatible with human existence is exceedingly extensive; for we find man inhabiting not only the hot burning plains of the tropics, but the frozen dreary and desolate regions of the poles. The experiments of Tillet, Fordyce, Delaroche, Blagden, Berger, &c., show that for a short time, man is capable of enduring a temperature far surpassing that ever reached at the hottest part of the earth's surface. In these experiments air heated even to 325° Fahr. was breathed, for some minutes, without any great inconvenience. The immediate effects were, a considerable increase in the rapidity of the pulse, and excessive perspiration, accompanied by considerable fatigue.

On the other hand, man can live for months (as they, for example, who have passed a winter in the polar regions,) in an air so cold as to freeze mercury.

It has been clearly shown that the effects of heat are manifested both upon the organic and the animal functions. On the former, they are seen in the increased activity of the circulatory and respiratory functions ; on the latter, they are followed by sensations of languor and fatigue.

The *functional* effects of heat are most distinctly marked upon the skin and the liver. The skin becomes changed in its appearance; and assumes either a dark or a yellow tinge, which may be attributed to an increase of the biliary secretion. The liver is stimulated to greater activity; the secretion of bile is greatly augmented, and its sensible properties are considerably changed. This high degree of functional excitement is frequently attended with inflammation of the organ.

The close relation observed between the skin and the liver, both in its physiological and pathological conditions, have induced some authors to speak of a " cutaneo-hepatic sympathy." (*Johnson on Tropical Climates.*) But while the skin and liver are thus *excited* by high and long continued atmospheric heat, the *lungs and kidneys act with diminished energy.*

*Cold, in its primary effects*, is in direct contrast with heat. The first is as decidedly sedative as the last is stimulating. Cold, continued for a length of time, diminishes the activity of all the functions, and reduces the being exposed to its influence, to a state resembling the sleep of hybernating animals. If continued still longer, it produces a state much resembling intoxication, followed by an excessive and overwhelming drowsiness, which is frequently the prelude to a sleep that terminates in death.

Equally contrasted are the *secondary* effects of heat and cold, if neither be carried so far as to derange the functions. Thus, while the excitement of heat is followed by languor and debility, the temporary depression from cold is succeeded by reaction, and a feeling of increased vigor. Cold, then, when moderate in degree, favors the activity both of the organic and animal functions—especially respiration, circulation, and nutrition—and it is followed by an increased development of animal heat.

This is not the place, nor have I time to dwell upon calorification. It is sufficient to make the statement, which is satisfactorily sustained by facts, that it can be referred to the action of no single organ, but is the result of a chemico-vital action, going on in every part of the system, by the combination of oxygen, taken in through the lungs, with the different tissues of the economy. It may be looked upon as the combustion of all the solid and fluid elements of the body. Hence every thing, such as good nutrition, regular respiration and circulation, that tends to increase the fuel, increases to a correspondent degree the development of animal heat. In speaking of cold as contributing to the development of animal heat, it is important to remember that it operates in an indirect manner, viz., 1st. By condensing the atmosphere, and thus furnishing for respiration a larger proportion of oxygen in a given time. 2d. By exciting to exercise, in order to resist the depressing influence of cold—thus increasing the circulation and respiration. 3d. By increasing the appetite for nutritive food—especially fatty matters—thus affording a larger amount of "heat generating material."

The *direct* operation of cold is to diminish the animal heat; and if continued in a great degree for a length of time, it may arrest its development by inducing a *torpor of the nervous system*, and thus enfeeble both the respiration and the circulation. The *direct* operation of heat, on the other hand, is not only to excite the functions generally, but also to *increase* the animal heat.

In estimating the influence of temperature upon the human frame, we should study its *comparative effects*, for we find the same climate producing totally different impressions upon its inhabitants, according to their race and prior climatic habits.

This is most strikingly exhibited in the following example from Andral, (*Dict. de Méd. et de Chir. pratique, art. Acclimatement.*) He states that the middle and higher portions of the island of Ceylon are inhabited by Europeans and negroes: the former, being exposed to a climate much hotter than that to which they were accustomed, die, in great numbers, of dysentery or hepatitis; while the latter, subjected to more cold than in their native climate, are rapidly carried off by pneumonia or phthisis.

Another element going to make up the complex problem of climate, is the *hygrometric* state of the atmosphere. This causes modifications in the human system, evinced by a striking difference in the external appearance, muscular development, activity, &c., of the inhabitants of a dry and hot, compared with those of a moist and sultry climate. For instance, the inhabitants of the sandy plains and deserts of Arabia and Africa, are thin and spare, but active: while those of the lower and moister countries as about the Nile, Niger, &c., exhibit large and gross frames, with a development of adipose and cellular tissue. These differences did not escape the attention of Hippocrates. In the valuable treatise already referred to, he describes the inhabitants of the Phasis, whose country is fenny, warm, humid, and wooded, and where copious and excessive rains occur at all seasons. "They drink the hot and stagnant waters both when rendered putrid by the sun and when swollen by the rains. The Phasis is the most stagnant of all rivers, and runs the smoothest; all the fruits which spring there are unwholesome, of feeble and imperfect growth, owing to the redundance of water, and on this account they do not ripen, for much vapor from the waters overspreads the country. For these reasons the Phasians have shapes different from those of all other men; for they are large in stature, and of a very gross habit of body, so that not a joint or vein is visible; in color they are sallow, as if affected with jaundice." They are naturally languid in supporting bodily fatigue. (Vol. 1, p. 209, Adams' edition for the Sydenham Society.)

Climate is also greatly modified by the *elevation* of a country above the level of the sea. The differences thus produced are very striking. In Mexico, for example, we have every variety of climate. Commencing with the low lands about Vera Cruz, which give origin to tropical fruits and tropical diseases, we gradually rise from district to district, with its diminished temperature, till we reach the city of Mexico, around which we find the temperature and productions of northern Europe. So with the regions of the Andes: travelling from the countries at their base, immediately under the Equator, we have variations from the scorching heat of a tropical sun, to the perpetual snows of Polar regions.

In all these elevated situations the atmospheric pressure is of course lessened. Hence we find that they give rise to an acceleration of the circulation, a tendency to pulmonary congestions, dyspnœa, and even to hemorrhages, in persons accustomed to inhabit situations nearer the level of the sea. In addition to the rarefaction of the air, these places are subjected to great and sudden changes of temperature, to excessively cold and violent winds, to the accumulation of dense fogs, &c.; and thus they exert upon the human frame many of the deleterious effects of Polar regions.

For a full, beautiful and satisfactory exposition of these effects, see "Traité d' Hygiéne Publique et Privée, par Michel Lévy." Tome 1, p. 367–380.

We next approach to a consideration of those climatic changes effected by the exposure of a country to particular winds. This also claimed the observation and attention of Hippocrates.

In the case of the Phasis, already alluded to, he particularly ascribes many of the peculiarities of its climate to the long continued effects of warm, southern winds. So, also, in a subsequent part of the same treatise, (p. 220,) when describing the different people of Europe, he says, "Such as inhabit a country which is mountainous, rugged and elevated, are naturally of an enterprising, warlike disposition, and have no litttle of the savage and ferocious in their nature." On the other hand he remarks, "Such as dwell in places which are low-lying, abounding in meadows, and ill ventilated, and who have a larger proportion of hot, than of cold winds, and who make use of warm waters—these are not likely to be of large stature, nor well proportioned, but of a broad make, fleshy," &c. "Courage and laborious enterprise are not naturally in them." With the region last described, is contrasted that inhabited by the Scythians, which Hippocrates (p. 213) tells us, "lies under the northern bears; and consists of plains, high-lying and naked, and not crowned by mountains." "The winds blowing from the hot regions of the earth do not reach them, or but seldom, and with little force; but the winds from the *north* always blow, congealed as they are, by the snow, the ice and much water."

The same description equally applies to the Tartars, &c., of Northern Asia, who inhabit a country which descends from the Himalayah mountains to the North Sea. How striking the contrast between the inhabitants of this region and those upon the southern declivity, running down to the Indian ocean, and of course exposed to the warm winds of the south.

Differences of nearly equal importance are observed between countries continually exposed to eastern, and those exposed to western winds.

The nature of the soil, the qualities of the water drunk by the inhabitants, and the extent of wooded lands, are also features not to be overlooked in our estimate of climate.

It must be quite clear, even from this necessarily brief and imperfect sketch, that mere temperature, or the degree of heat or of cold, can convey but a very inadequate idea of the character of the seasons and climate of a country. Nor, in estimating even the temperature of any region, can we trust alone to its latitude, or its distance from the equator; for the enquiries of Humboldt and others, clearly show, that the "Isothermal lines" do not correspond with those of latitude, nor are they parallel to each other. Nor are the "Isotheral lines," or those of equal summer, parallel to the "Isocheimal lines," or those of equal winter; nor either of these with the "Isothermal lines." A forcible illustration of this fact is presented in the close resemblance of the winter climate of parts of Devon and Cornwall, in England, to that of central Italy, although they are separated by several degrees of latitude, and differ so much in their summers.

[As a still more striking exemplification of this truth, I would ask attention to the following extract from a speech delivered by Mr. Benton, in the United States Senate. Speaking of the climate of New Mexico, he says, "Humboldt thus describes it: 'New Mexico, though placed under the same latitude with Syria and Central Persia, has a climate eminently cold. It freezes there in the middle of the month of May, near to Santa Fee, and a little further north (under the parellel of the Morea,) the Rio del Norte is covered, sometimes several years in succession, with ice so thick that horses and carriages pass on it.' *Essay on New Spain*, vol. i. p. 103.

'The environs of El Paso are a delicious country, which resemble the most beautiful parts of Andalusia. The fields are cultivated in corn and wheat. The vineyards produce excellent wines. The gardens contain in abundance all the fruit trees of Europe.' (Vol. iii. p. 306.)

"Humboldt," continues Benton, "is right, and recent travellers now confirm what he wrote in 1804. It was at the head of the valley of the Del Norte, some three degrees north of Santa Fee, that Col. Fremont suffered his great disaster—had to struggle through snows above the heads of men and horses, and found it a relief to tread the river, solid with ice, for a road. At Santa Fee, the 20th of February, it was winter; eight days afterwards, on the

Rio Abajo, half way to El Paso, and having descended 2600 feet, and still 1200 feet above the level of El Paso, it was spring, the farmers plowing and seeding, the early fruit trees in bloom, and the air so mild that he camped out at nights without tents, though in a settled and hospitable country."]

Having noticed some of the peculiarities of different climates, and having shown that they modify more or less the constitutions of all who come within the sphere of their continued action, the question naturally arises : can man, with his constitution thus moulded by his native climate, remove to a foreign country without risk of injury to his health, or of suffering from disease ? We answer, without hesitation, in the negative.

The history of European colonization and conquest, especially in the East and West Indies, and in Northern and Central Africa, exhibits a frightful loss of life, owing to the changes of climate and the difficulty of acclimation. Even in our own country, we have seen with what a great sacrifice of life have been accomplished, first the settlement of our Atlantic, and afterwards that of our Southern and Western States.

Yearly, the natives of the Northern States, who in the pursuit of commerce, or of pleasure, make a sojourn in the Carolinas or in Louisiana, pay the penalty of disease, and too frequently of life itself.

A knowledge of these and similar facts, must make us slow to credit, at least to the extent generally entertained, the opinion that man possesses the power of adapting himself to *all changes* of climate. A recent writer, M. Boudin, in an article entitled "Etudes dé Pathologie Comparative,"* formally protests against such an opinion, "a belief of which, he thinks, not resting upon any experimental basis, could only have originated from what has been observed of a fraction of humanity represented by what we call the Caucasian race." "From the earliest times to our own day, we see the European fail in all his attempts at acquiring a permanent hold upon the land of Egypt; where, also, the Negro and the Mameluke are shown to be incapable of procreating beyond the third generation. In Corsica, the Italian termination of family names proves, of itself, the inability of the French to establish their stock upon that island. Where, in the north of Africa, do we find the descendants of the Romans and the Vandals ? Why, in America,

* Ann. d' Hyg. and de Med. Leg., 1849.

continues Boudin, after passing the 36th degree of latitude, do we meet with slavery everywhere, unless where the elevation of the land mitigates the deleterious influence of an excessively increased temperature? The height above the ocean which gives protection to the life of a European, in hot climates, becomes fatal to the negro. Out of 53 black soldiers posted at Ninera Elia, in the island of Ceylon, at 6200 feet above the level of the sea, 15 died before the end of the year. In the earliest times, despotism made use of exile into countries alien to their nature, for the destruction of different nations. With this view, after the destruction of Jerusalem, were a great number of Jews sent to Sardinia, on the occasion of whose exile Tacitus makes the following reflection: 'Et si ob gravitatem cœli interissent, vile dammum.' After the war of the Morea, Mehemet Ali, wishing to get clear of the undisciplined Arnouts, sent them to the shores of the Red Sea, *where in a few years,* 1800 *men were reduced to* 400, *by the mere influence of the climate.*" A forgetfulness or an ignorance of the incompatibility of certain races with particular regions of the earth, has caused an immense loss of life and the failure of the most costly expeditions. "Thus, in 1817, a negro regiment, placed in garrison at Gibraltar, was almost entirely destroyed by pulmonary consumption. In 1841, the expedition to the Niger failed, perhaps owing to the bad selection of the crews of the vessels. In *three weeks* after having entered the Niger, 130 *out of* 145 *white men were attacked with fever, and* 40 *sank under it.* Out of 158 *negro sailors,* on the other hand, born in America, in the West Indies, or on the coast of Africa, 11 *only* were assailed by fever—but 9 of the cases were fatal. Thiers, in his history of "the Consulate and the Empire," shows the dreadful loss of life among the French troops employed in the invasion of St. Domingo. "But 7000 or 8000 men remained out of an army of 32000; 15000 *were carried off in two months.* At the same time, in which Toussaint l'Ouverture, the sinister prophet who had foretold and longed for these disasters, died of cold in France, a prisoner at the fort of Joux, our soldiers sank under the piercing rays of a destroying sun." It would be easy to multiply examples to the same effect; all going to show the heavy tax paid by those who adventure into remote lands, and into climates differing from that of their nativity. Not only are immense numbers carried off by disease, but the survivors are reduced below their former standard of bodily strength and mental vigor, and thus are made an easy prey to disease; and in some places it has been doubted whether the engrafted stock could last many generations. Twining asserts (Johnson op. cit.) that, in the delta of the Ganges, such is the influence of its climate, the unmixed European race becomes extinct at the third generation.

Notwithstanding, however, all these instances, we are forced to admit that the human constitution undoubtedly possesses the power of accommodating itself to new, and oftentimes to the most opposite, climatic influences; and this not merely in the case of a few individuals, but of entire communities, and even of great nations. A notable example is afforded in the case of the Jews who are scattered over the habitable globe—all of them retaining features which mark their common origin. But still, if we compare a Dutch with a Spanish Jew, and these again with a Jew of Malabar, we observe a striking difference in their appearance; and also find them exhibiting the shades of complexion, the color of the skin, and the general external development, indicative of the climatic influences to which they are respectively subjected, and which approximate them to the natives of these different countries. We see clearly, that though the race is unmistakeably continued, it has undergone changes, only to be attributed to the influence of a new and foreign climate.

It now remains for us to enquire into the changes by which the process of acclimation is accomplished. First we shall speak of those effected by a removal from a cold to a hot climate, or in other words, SOUTHERN ACCLIMATION.

We have seen that, *in a cold climate*, man's circulation and respiration must be active; that his nutrition must be good, in order to supply the materials for the development of heat sufficient to protect him from the injurious influence of the cold atmosphere by which he is surrounded. In this condition, he is carried to a tropical climate, where the active evolution of heat is not only unnecessary, but injurious; he arrives with all his organs actively discharging their respective functions; developing a quantity of heat far exceeding the demand. Were it not for the abundant perspiration that accompanies this functional excitement, the new-comer would be exposed to the most imminent danger.

It is owing to this fact that, as experience shows, the acclimation of delicate, weak, or old persons, is more easily accomplished than in the case of those possessing strong, energetic constitutions, and with a tendency to plethora.

The stranger, in a *hot climate,* is subject to the following changes: His circulation becomes accelerated; he suffers extremely from excessive heat; is subject to the most distressing restlessness, in his being for a time utterly unable to procure sleep; and he exhibits a strong tendency to local congestions, particularly to the intestinal canal, brain, liver and skin. All strangers, however, are not thus affected; nor can we give any fixed, invariable description that will be applicable to all. We see some affected with a trival indisposition, which soon disappears, and they are well. Some, after continuing for a few days slightly indisposed, are attacked by an affection of brain, liver or intestinal canal, by which they are frequently carried off: others, without any previous warning, are suddenly seized with a fatal inflammation of these organs. The greater number, however, at first, seem to be but slightly affected by the change of climate; but, by degrees, their bodies are emaciated, their strength declines, languor and debility are depicted upon their countenances, and they, unconsciously, becomes victims of chronic diseases of the liver and intestinal canal.

From what has been before stated, we are prepared to learn that countries in the same latitude, do not exert the same influence upon strangers. We find that these countries, owing to peculiar situation, temperature, and many other circumstances, give origin to different diseases, and thus operate in a different degree upon new comers.

It would carry me too far from the immediate subject of this essay, and occupy too much time, to enter fully into this subject; hence I must content myself by making the general statement, that countries of the same latitude, however different may be their endemic diseases, and however different their primary effects, produce ultimately about the same mortality. Though many examples might be quoted, the following from Andral (op. cit.) affords a good illustration of this point. " Out of *one thousand British* troops sent to Jamaica, it was found that *four to five hundred of them perished during the first eight months;* while out of an

*equal number* sent to Madras during *the same period, only sixty to seventy died.* But this disproportion diminished as their sojourn continued, so that at the end of two years the *number of deaths, in the two stations, was nearly equal.*" In the former case, they were carried off by yellow fever, or some similar disease, which rarely occurs more than once to the same individual; and as the climate is mild, they that escape this attack, afterwards enjoy good health; whereas, in the latter place, near Madras, they are exempt from yellow fever and other such epidemics, so that few die at first, but after remaining awhile, exposed to the climate, with its atmospheric extremes, their constitutions become enfeebled and broken down, and they are carried off in great numbers by dysentery, or some disease of the stomach or liver.

(To be continued.)

